# Atypical Porcine Pestivirus (APPV) as a New Species of *Pestivirus* in Pig Production

**DOI:** 10.3389/fvets.2019.00035

**Published:** 2019-02-21

**Authors:** Igor Renan Honorato Gatto, Karina Sonálio, Luís Guilherme de Oliveira

**Affiliations:** School of Agricultural and Veterinarian Sciences, São Paulo State University (Unesp), São Paulo, Brazil

**Keywords:** atypical porcine pestivirus, congenital tremor, pestiviruses, pig production, pre-weaning mortality

## Abstract

The genus *Pestivirus*, which belongs to the family *Flaviviridae*, includes ssRNA+ viruses responsible for infectious diseases in swine, cattle, sheep, goats, and other domestic and wild animals. Recently, several putative pestiviruses species have been discovered and characterized in mammalian species (giraffe pestivirus, antelope pestivirus, HoBi virus, Bungowannah virus, and Linda virus); one of these is a genetically distinct pestivirus, named atypical porcine pestivirus (APPV), discovered using the next-generation sequencing technology. APPV has been detected in piglets with congenital tremor (CT) from four different continents, including North America, South America, Europe, and Asia. There is strong evidence that experimental inoculation and *in field* outbreaks involving APPV induce CT in piglets. Additionally, splay leg (SL) syndrome has been observed concurrently with CT, and it was induced by APPV in experimental studies and some field cases. Animals with a persistent and/or chronic infection condition can shed the virus over time. Viral-RNA is frequently detected in different tissues from CT-piglets; however, high loads of APPV are detected most consistently in central nervous tissue. Moreover, the APPV genome has been recently detected in semen and preputial swabs from boar studs, as well as in serum and tissue samples from wild boars and domestic adult pigs, all known to be clinically healthy. Phylogenetic analysis revealed that the APPV sequence (complete or partial polyprotein) exhibits high genetic diversity between viral strains detected in different countries and formed independent clusters according to geographic location. Additional studies are needed to evaluate the molecular detection and sero-prevalence of APPV around the world. Lastly, more research is needed to understand clinical presentations associated with APPV infection, as well as the economic losses related to the virus in pig production worldwide.

## Introduction

Piglet pre-weaning mortality is a major problem in pig farms around the world. On average, pre-weaning mortality rates of live-born piglets can be as high as 23% and starvation and crushing are the main causes of death (1). Different etiologies may be involved in piglet pre-weaning mortality including atypical porcine pestivirus-associated congenital tremor (CT) (2–4).

Historically, CT was first reported in the literature 97 years ago, when Kinsley (5) described “dancing pigs.” Subsequently, it was characterized as tremors of intent that ceases when piglets are at rest (6). The syndrome is classified into five types according to the etiology (Type AI–AV) (Table 1); however, most contemporary CT outbreaks had been attributed to an unidentified virus, Type AII (7, 8).

**Table 1:** Etiology of congenital tremor types in piglets.

	**Congenital tremor type**				
	**A-I**	**A-II**	**A-III**	**A-IV**	**A-V**
Etiology	Classical swine fever virus	Atypical porcine pestivirus	Genetic sex-linked recessive	Genetic autosomal recessive	Chemical trichlorfon
Breed	All	All	Landrace	Saddleback	All
Affected litters	High	High in gilts Low in sows	Low	Low	High
Mortality of CT-piglets	Moderate-high	Low-moderate	High	High	High

Since atypical porcine pestivirus (APPV) was first identified in 2015 (9), several studies have linked this new *Pestivirus* with the occurrence of CT in newborn piglets. It was usually described as a temporary condition, lasting several weeks to months, and characterized by tremors of the head, limbs, and body, varying in severity and intensity. However, the clinical signs were reduced or absent during inactivity or sleep (2, 3, 10).

In general, CT is not the cause of death in affected piglets; however, their survival may be threatened because of inadequate colostrum, or milk intake, leading to severe growth retardation and death by starvation or crushing due to impairment of evasive actions (2, 7). Furthermore, APPV is capable of inducing neurological disorders, such CT, increasing piglet pre-weaning mortality and reducing reproductive performance in affected pigs (2–4, 10).

Although the impact of most exotic diseases in animal production and global economy is known (11), a more comprehensive understanding of the epidemiology, genetic variability, and economic losses associated with the role of APPV in pig production is required.

## Etiology

Pestiviruses are highly variable RNA viruses causing economically relevant diseases in domestic animals. The genus *Pestivirus*, which belongs to the family *Flaviviridae* (ssRNA +), includes 11 recognized species: *Pestivirus* A (bovine viral diarrhea virus type 1), *Pestivirus* B (bovine viral diarrhea virus type 2), *Pestivirus* C (classical swine fever virus), and *Pestivirus* D (border disease virus), *Pestivirus E* (pronghorn pestivirus), *Pestivirus F* (Bungowannah virus), *Pestivirus G* (giraffe pestivirus), *Pestivirus H* (Hobi-like pestivirus), *Pestivirus I* (Aydin-like pestivirus), *Pestivirus J* (rat pestivirus), and *Pestivirus K* (atypical porcine pestivirus) (12).

Additionally, three atypical pestiviruses have been characterized in pigs: Bungowannah virus (causing myocarditis), APPV and Linda virus (causing lateral shaking) (9, 13, 14). Nevertheless, several reports (experimental conditions or field cases) have demonstrated that APPV is a prominent cause of APPV-CT type II in newborn piglets around the world (2–4, 10, 15). However, no studies have provided an efficient protocol for APPV isolation in cell culture (3, 9, 16, 17), and Koch's postulate couldn't be established.

Further characterization of APPV, as well as other porcine pestiviruses linked to severe clinical diseases in pigs, is needed (18, 19). Remembering that, the continued expansion of the genus *Pestivirus* and its high genetic diversity constitute a worldwide concern.

## Geographic Distribution

**Figure 1:**
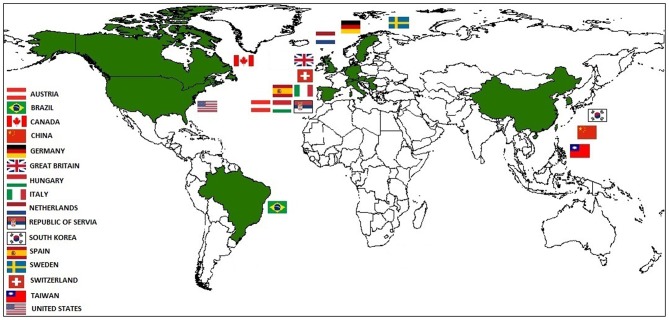
Geographic distribution of Atypical Porcine Pestivirus (APPV) around the world. The geographic information system ArcGIS 10.5.1 was used to generate the map.

APPV has been detected in four different continents, including North America, South America, Europe, and Asia, and in countries such as the United States (first report) (9, 10), the Netherlands (2), Germany (15, 16), Sweden (20), Spain (2, 21), Austria (3), China (22), South Korea (23), Brazil (4, 24, 25), Canada (26), Hungary (27), Great Britain (28, 29), Italy, the Republic of Serbia, Switzerland, and Taiwan (29). Considering all these reports, APPV has wide distribution in the world (Figure 1). Postel et al. (29) have suggested that APPV must be regarded as a pig pestivirus of likely worldwide relevance.

## Epidemiology

APPV-associated CT has been reported to be more prevalent in litters of gilts than sows (2, 4), suggesting that the immune status of the dam is likely the key in disease development in piglets (4). During outbreaks, CT morbidity varies within and between litters; a few pigs in one or all pigs in several litters may be affected (2–4, 10). Overall, CT is observed in both males and females (2) and its prevalence within the litters ranges from < 10 to 100% (2–4, 10). Moreover, CT severity in piglets seems to vary within litters (10). Several recent studies have linked APPV with CT occurrence in piglets and sporadic detection in domestic and wild boars. Detailed information regarding APPV studies can be found in Table 2.

**Table 2:** Summary of atypical porcine pestivirus studies.

**Year**	**Country**	**Serology**	**Strength(s)**	**References**
2015	United States	+	Virus discovered by next-generation sequencing	(9)
2016	United States	–	Experimental inoculation in fetuses (45 and 62 days of gestation)	(10)
2016	Netherlands	–	Experimental intramuscular inoculation (32 days of gestation)	(2)
2016	Germany	–	Detection of APPV genome by fluorescent *in-situ* hybridization/Detection in adult domestic pigs/Virus isolation was attempted (failed)	(15)
2016	Germany	–	First indication of a cell culture isolate is provided/Detection in adult domestic pigs	(16)
2017	Austria	+	Persistent infection condition was suggested/Virus isolation (inefficient)	(3)
2017	China	–	Viral strains showed highly genetic diversity	(30)
2017	China	–	Suggested APPV origin and dissemination/Virus isolation attempted (failed)	(22)
2017	Great Britain	–	First detection in the country	(28)
2017	Spain	+	Retrospective study (virus was identified at least since 1997)/Detection in adult domestic pigs	(21)
2017	Switzerland/China/Great Britain/Germany/Italy/Republic of Serbia/Taiwan	+	Geographically wide distribution of genetically highly variable APPV and high APPV genome detection	(29)
2017	South Korea	–	First detection in the country	(23)
2018	China	–	Suggested a novel APPV strain in China	(31)
2018	Brazil	–	First detection in Brazil/Formalin-fixed paraffin-embedded samples were used	(4)
2018	Brazil	–	Sequencing and analysis of the partial NS5B gene	(24)
2018	Brazil	–	High lethality and coinfection with porcine teschovirus (PTV)	(25)
2018	United States	–	Detection in semen, preputial swabs, and preputial fluids from boar studs	(32)
2018	China	–	Identification and characterization of two possible strains	(33)
2018	Germany/Republic of Servia	+	First detection in wild boars/Detection of APPV-antibodies in wild boars from the Republic of Serbia	(34)
2018	Hungary	+	First detection in this country/Distinct lineages were reported, suggesting multiple introduction events of the virus	(27)
2018	Canada	–	First detection in the country	(26)
2018	Spain	–	Detection in wild boars/Low prevalence	(35)
2018	Brazil	–	APPV-associated with pathological lesions	(36)

Adult domestic pigs (15, 16, 21) and wild boar are susceptible to APPV infection (34, 35). Recently, APPV was detected in boar preputial fluids (2) and semen (3); both sample types came from boars that had clinical signs consistent with CT at birth. Still, APPV has been detected in semen, preputial swabs, and preputial fluids from commercial boar studs in the United States (32) and it is highly improbable that these boars had CT at the time of birth, suggesting that either transiently infected or persistently infected (PI) boars with no clinical history of CT could be shedding APPV in semen.

A retrospective analysis of cerebellum samples from Germany and another retrospective study with fresh and formaldehyde-fixed paraffin-embedded tissue samples from Hungary showed the presence of APPV in CT-affected piglets from over a decade ago (15, 27). Similarly, a study from Spain confirmed the presence of APPV nearly two decades prior to its first discovery (21).

Wild boar is also susceptible to APPV infection, although its role in the virus epidemiology is unknown (35). Limited information regarding APPV route of transmission, ecology, pathogenesis, carriage, spread, and epidemiology is available. However, piglets presenting with CT, surviving CT syndrome piglets, boars without CT (at birth), and clinically healthy adult domestic pigs, can shed moderate to high loads of virus, playing a relevant role in virus epidemiology; similar to a chronically and/or PI animal (2, 3, 32, 34).

Regarding to diagnosis, Postel et al. (17) described the presence of viral genome in serum samples with different levels of antibodies, suggesting a degree of antibody protection; and, samples with absence of antibodies and viral genome loads, indicating acutely or PI animals. Similarly, Muñoz-González et al. (21) also suggested the PI condition, which could help the APPV spread. On the other hand, the presence of systemic levels of type I Interferon in newborn piglets could lead to the activation of the immune system by APPV (21). Based on these results, more research is needed in order to better understand the role of the innate immune system response to APPV infection.

## APPV Pathogenesis and Pathology

The newly discovered APPV (9) is capable of inducing neurological disorders, reducing reproductive performance, and increasing pre-weaning mortality (2–4, 10). Two independent research groups have experimentally reproduced CT using an inoculum containing APPV and observed a subset of piglets with concurrent splayleg (SL) (2, 10) and that the affected CT-litters presented as weak piglets with an abnormal posture (2). Field studies have also reported the occurrence of SL in litters with CT sporadically affecting the same piglet (3, 4). SL prevalence ranged from 6 to 55% within the affected CT litters (4).

APPV has a wide distribution in tissue samples, excretion and secretion fluids (2, 10, 15, 21, 22, 32). According to Gatto et al. (4), central nervous and lymphoid tissues appear to be suitable sites for viral replication; however, the cerebellum was the most consistently positive sample type from CT piglets and could constitute a target for APPV replication. Although a specific target of replication has not been determined (10), this may suggest that viral replication occurs systemically and has a predilection for certain types of tissues. However, the primary replication sites remain unknown. The histological findings of a number of studies are described in Table 3.

**Table 3:** Histopathological and histochemical findings from Congenital Tremor (CT) cases.

**Country**	**Histopathological and histochemical findings**	**References**
Germany	No significant findings in the central and peripheral nervous system, as well as skeletal muscles. Luxol fast blue staining revealed mildly reduced staining intensity accentuated in the lateral white matter of the spinal cord	(15)
Austria	Vacuoles in cerebellar white matter; moderate hypomyelination in the white matter of the cerebellum and thoracic spinal cord; detection of oligodendrocytes; and increased staining intensity	(3)
Brazil	Moderate vacuolization of the white matter of the cerebellum and brain stem. Luxol fast blue staining did not reveal a decrease in the amount of myelin in the cerebellum; however, mild myelin loss was noted in the white matter found in the spinal cord and sciatic nerve	(24)
Brazil	Luxol fast blue staining revealed evidence of myelin vacuolization with the formation of digestion chambers. These chambers were of different sizes, observed in the white matter of the cerebellum, brainstem, and spinal cord. Severe secondary demyelination, with either a complete absence or an inadequate amount of myelin, in areas in both the white and gray matter of the spinal cord and brainstem, with mild secondary demyelination in the cerebellum	(25)
China	No significant findings	(30)
Canada	Luxol fast blue staining revealed an important loss of myelin from the periphery of the thoracic spinal cord, more severe in the lateral and ventral funiculi	(26)
Brazil	Neuronal necrosis, gliosis, and neuronophagia with satellitosis particularly at the cerebral cortex and to a lesser extent at the spinal cord, white matter demyelination of the cerebrum and spinal cord, Wallerian degeneration of the spinal cord, and necrosis of Purkinje cells of the cerebellum. The immunohistochemistry revealed proliferation of glial fibrillary acidic protein (GFAP) cells and fibers were more severe and widespread in piglets infected by APPV	(36)

## Impact on Pig Production

The economic relevance of an APPV-outbreak loss in pig production worldwide remains undetermined; however, it is estimated that the number of weaned piglets per sow decreases by > 10%, affecting reproductive performance. Additionally, mortality increased up to 30%, when CT-affected new-born piglets died of malnutrition in a farm in Austria (3). de Groof et al. (2) reported 26% mortality in CT-litters affected by APPV, with 60% of these deaths attributable to CT in one farm. Additionally, they showed that under experimental conditions, the affected CT-litters presented weak piglets with an abnormal posture (bent back [kyphosis] and ears on the neck) (2). The SL syndrome has been observed concomitant with some CT cases (2–4, 10), limiting the locomotion of the CT-piglets and increasing the percentage of crushing.

In China, Shen et al. (33) reported a mortality rate of 60% in CT-piglets and Dessureault et al. (26) reported an average mortality rate of 24.6% in CT-litters in Canada. According to Gatto et al. (4) the case fatality of affected CT-piglets in Brazilian pig farm production was 30%. Early data from the United States, Germany, Italy, China, and Taiwan have suggested a relatively high abundance (2.3–22%) of APPV genomes in apparently healthy pigs (9, 15, 16, 29). In addition, Gatto et al. (32) detected APPV genomes in semen (up to 34% prevalence), preputial swabs (up to 23%), and preputial fluids (up to 28%) from commercial boar studs in the United States, which could play an important epidemiological role in virus transmission route and spread. Thus, artificial insemination could constitute a potential APPV transmission route and should be considered an important factor when developing and implementing biosecurity measures to prevent APPV-infections.

The impact of APPV infection on pig production seems to be indirect, since the mortality of CT piglets is the only loss reported so far, and it is mainly attributed to secondary factors. So, further studies evaluating its impact on pig production should be performed in order to estimate the direct loss in affected piglets, or suggest that the indirect loss could be related to the depletion of the immune system, such as what is reported in other pestiviruses.

## Atypical Porcine Pestivirus Genome

Since 2015, 20 complete APPV polyproteins from six countries (Table 4) have been submitted to the “GenBank” database. The virus genome of *Pestivirus* K species is ~10.8–11.5 kb. The APPV genome has ~25–28% pairwise identity to known pestiviruses and 68% pairwise identity to a recently partially characterized *Rhinolophus affinis* pestivirus, placing both viruses in a highly divergent lineage of pestiviruses (9).

**Table 4:** List of complete atypical porcine pestivirus genomes.

**Number**	**Genbank accession**	**Country**	**Year**	**Lenght (bp)**
1	KR011347	United States	2015	11.276
2	KU194229	United States	2015	11.545
3	KU041639	Germany	2015	10.908
4	LT594521	Germany	2016	11.467
5	MF167290	Germany	2017	10.908
6	MF167291	Germany	2017	10.908
7	KX929062	Netherlands	2016	11.561
8	KX778724	Austria	2016	11.535
9	KX950761	China	2016	11.043
10	KX950762	China	2016	11.043
11	KY475592	China	2017	11.304
12	KY475593	China	2017	11.464
13	KY612413	China	2017	11.043
14	KY624591	China	2017	11.466
15	KY652092	China	2017	11.475
16	MF167292	China	2017	10.815
17	MF377344	China	2017	11.556
18	MG792803	China	2018	11.526
19	MH102210	China	2018	11.534
20	MF979135	South Korea	2017	11.247

**Figure 2:**
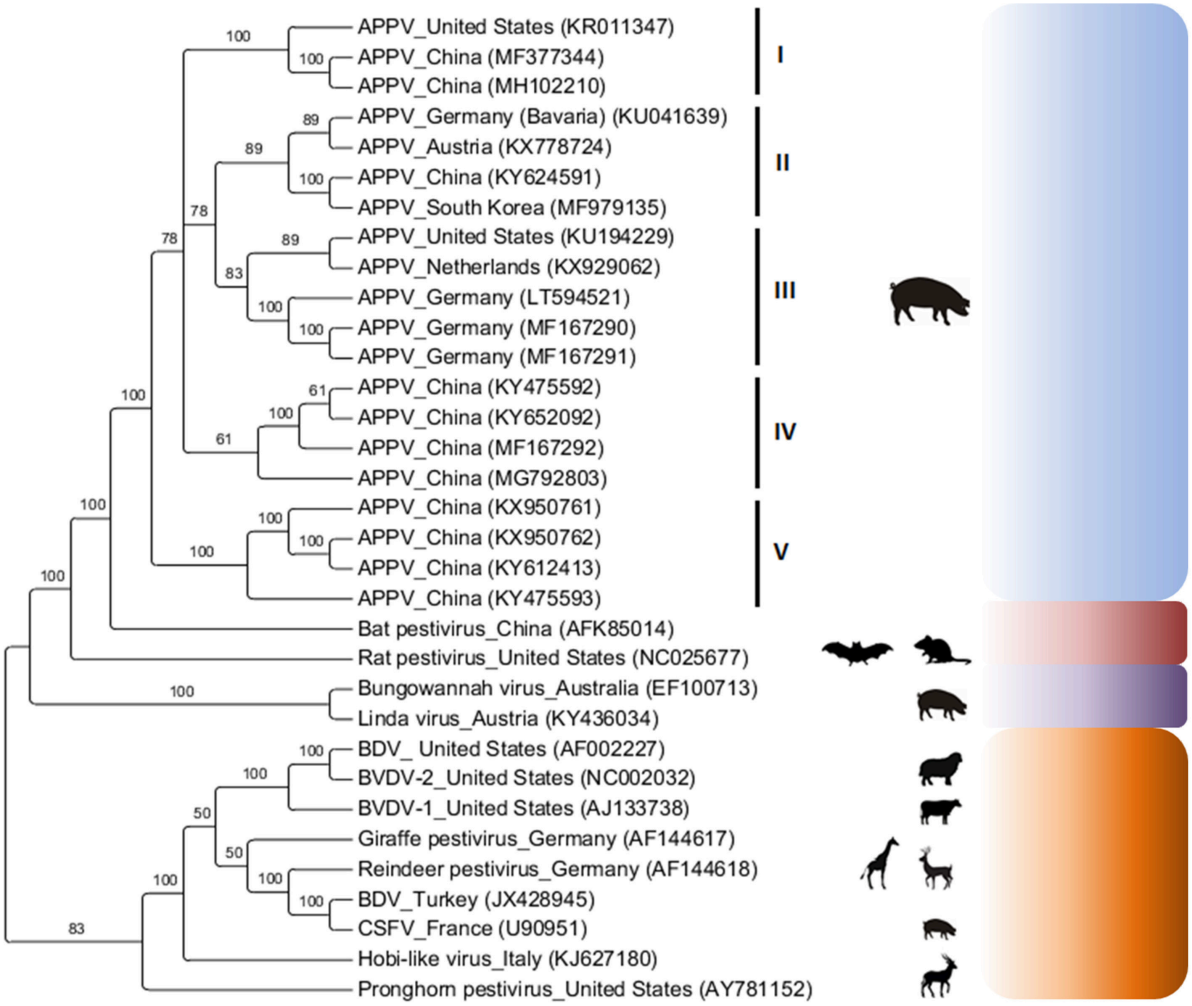
Phylogenetic analysis of the atypical porcine pestivirus complete polyprotein (13,695 bp after alignment). Analysis was based on the Bayesian method and the GTR + I + G4 evolutionary model using MrBayes 3·1·2 (37) via CIPRES Science Gateway (38). The numbers on the nodes correspond to clade support values accessed with 10^6^ bootstrap replicates. Viruses of the genus *Pestivirus* (classical and atypical) were used as an external group.

Phylogenetic analysis has revealed that APPV sequences (complete or partial polyprotein) exhibit high genetic diversity between viral strains detected in different countries (4, 27, 29–31) and form independent clusters according to geographic location (Figure 2). Based on phylogenetic analysis of the Npro gene, different viral strains can be present in the same farm simultaneously and at different times (32).

A phylogenetic tree based on Bayesian analysis of 20 complete APPV polyprotein sequences and other pestiviruses demonstrated a monophyletic cluster topology for APPV. In addition, five distinct clusters were observed within the APPV clade (cluster I: North America and Asia, cluster II: Europe and Asia, cluster III: North America and Europe and clusters IV and V: Asia; Figure 2).

## Future Prospects

The economic impact of some pestiviruses in swine species is manifested by devastating losses worldwide. The high genetic variability of pestiviruses is the key point triggering practical consequences in epidemiology, diagnosis, control, and economic impact on livestock, especially in pigs.

Given the wide distribution of APPV and its genetic variability in different countries, studies regarding the epidemiology, ecology, pathogenesis, pathophysiology, transmission routes, and the impact of this virus on swine farms are necessary. Recent detection of APPV in semen from commercial boar studs has sparked further investigations to clarify whether APPV can be transmitted through artificial insemination or reproductive biotechnologies, commonly used in pig breeding, which might play a significant role in the dissemination of pathogens.

Another interesting epidemiological issue is the recent detection of APPV in wild boars, demonstrating the ability of the virus to infect domestic and wild pigs. To date, the role of this wild species in the epidemiology of APPV remains unknown and further research should be conducted with this species, as the world's population of wild boars is increasing. Moreover, the impact of this ecological imbalance on the maintenance and spread of pathogens could be devastating, as in the case of classical swine fever virus spread in Europe.

Based on the available data, we highlighted some key points related to APPV infection, such as: (1) adaptive immunity of gilts/and sows; (2) possible transmission of the virus by semen; (3) absence of a commercial vaccine. For this reason, we hypothesize and suggest a few insights that may have a positive impact on the control of the infection. Since the highest prevalence of CT-piglets occurs in gilts, we suggest that the immune status of the dam and the time of infection are the key factors related to disease development. So, we recommend the use of an acclimatization strategy for replacement gilts, similarly to the protocol used for *Mycoplasma hyopneumoniae* control. In addition, to reduce the risk of APPV transmission by semen, it is advised that batches used in artificial insemination protocols are previously tested for the presence of APPV genomes. Likewise, based on the high genetic variability of APPV and recent research about the development of a subunit vaccine against APPV based on the E2 protein (39), we strongly recommend the implementation of a feedback management in farms with CT-cases, strategy that could be used until the development of an efficient commercial vaccine. Therefore, additional epidemiological information is required in order to develop strategies of control and eradication of APPV in pig production.

Finally, even though the potential for intercontinental spread of some viruses and the impact of exotic and emerging diseases on worldwide pig production is known, we strongly recommend additional epidemiological studies that will provide current essential information regarding APPV and elucidate possible routes of entry, dissemination, and genetic evolution of APPV, as well as other viral agents. This data will aid in the active surveillance of pathogens considered exotic and/or emerging around the world (i.e., porcine epidemic diarrhea virus, transmissible gastroenteritis virus, porcine reproductive and respiratory syndrome virus, and African swine fever virus).

## Author Contributions

All authors listed have made a substantial, direct and intellectual contribution to the work, and approved it for publication.

### Conflict of Interest Statement

The authors declare that the research was conducted in the absence of any commercial or financial relationships that could be construed as a potential conflict of interest.

## References

[1] Kielland C Wisløff H Valheim M Fauske A Reksen O Framstad T. Preweaning mortality in piglets in loose-housed herds: etiology and prevalence. Animal (2018) 12:1950–7. 10.1017/S1751731117003536 29306344

[2] de Groof A Deijs M Guelen L van Grinsven L van Os-Galdos L Vogels W Atypical porcine pestivirus: a possible cause of congenital tremor type A-II in newborn piglets. Viruses (2016) 8:271 10.3390/v8100271 PMC5086607 27782037

[3] Schwarz L Riedel C Högler S Sinn LJ Voglmayr T Wöchtl B. Congenital infection with atypical porcine pestivirus (APPV) is associated with disease and viral persistence. Vet Res. (2017) 48:51. 10.1186/s13567-016-0406-1 28057061 PMC5217315

[4] Gatto IRH Harmon K Bradner L Silva P Linhares DCL Arruda PH. Detection of atypical porcine pestivirus in Brazil in the central nervous system of suckling piglets with congenital tremor. Transbound Emerg Dis. (2018) 65:375–80. 10.1111/tbed.12824 29393592

[5] Kinsley A Dancing pigs? Vet Med. (1922) 17:123.

[6] Stromberg MW Kitchell RL Studies on myoclonia congenita. I Review of literature and field investigations. Am J Vet Res. (1958) 19:377–82. 13533760

[7] Bolin SR Congenital tremors virus. In: Leman AD Straw BE Mengeling WL D'Allaire S Taylor DJ, editors. Diseases of Swine. London: Wolfe Publishing Ltd (1992). p. 247–9.

[8] Done S Williamson SM Strugnell BW Nervous and locomotor systems. In: Zimmerman JJ Karriker LA Ramirez A Schwartz KJ Stevenson GW, editors. Diseases of Swine. Ames, IA: Wiley-Blackwell (2012). p. 294–328.

[9] Hause BM Collin EA Peddireddi L Yuan F Chen Z Hesse RA. Discovery of a novel putative atypical porcine pestivirus in pigs in the USA. J Gen Virol. (2015) 96:2994–8. 10.1099/jgv.0.000251 26219947

[10] Arruda BL Arruda PH Magstadt DR Schwartz KJ Dohlman T Schleining JA. Identification of a divergent lineage porcine pestivirus in nursing piglets with congenital tremors and reproduction of disease following experimental inoculation. PLoS ONE. 11:e0150104. 10.1371/journal.pone.0150104 26909691 PMC4766193

[11] Waage JK Mumford JD. Agricultural biosecurity. Philos. Trans. R. Soc. Lond. B. Biol. Sci. (2008) 363:863–76. 10.1098/rstb.2007.2188 17761470 PMC2610114

[12] Smith DB Meyers G Bukh J Gould EA Monath T Scott Muerhoff A. Proposed revision to the taxonomy of the genus *Pestivirus*, family *Flaviviridae*. J Gen Virol. (2017) 98:2106–12. 10.1099/jgv.0.000873 28786787 PMC5656787

[13] Kirkland PD Frost MJ Finlaison DS King KR Ridpath JF Gu X. Identification of a novel virus in pigs - Bungowannah virus: a possible new species of pestivirus. Virus Res. (2007) 129:26–34. 10.1016/j.virusres.2007.05.002 17561301

[14] Lamp B Schwarz L Högler S Riedel C Sinn L Rebel-Bauder B. Novel *Pestivirus* species in pigs, Austria, 2015. Emerg Infect Dis. (2017) 23:1176–9. 10.3201/eid2307.170163 28628456 PMC5512468

[15] Postel A Hansmann F Baechlein C Fischer N Alawi M Grundhoff A. Presence of atypical porcine pestivirus (APPV) genomes in newborn piglets correlates with congenital tremor. Sci Rep. 6:27735. 10.1038/srep27735 27292119 PMC4904412

[16] Beer M Wernike K Drager C Hoper D Pohlmann A Bergermann C. High prevalence of highly variable atypical porcine pestiviruses found in Germany. Transbound Emerg Dis. (2017) 64:e22–e26. 10.1111/tbed.12532 27297961

[17] Postel A Meyer D Petrov A Becher P. Recent emergence of a novel porcine pestivirus: interference with classical swine fever diagnosis? Emerg Microbes Infect. 6:e19. 10.1038/emi.2017.5 28400592 PMC5457672

[18] Moennig V Becher P. *Pestivirus* control programs: how far have we come and where are we going? Anim Health Res Rev. (2015) 16:83–7. 10.1017/S1466252315000092 26050577

[19] Kirkland PD Read AJ Frost MJ Finlaison DS. Bungowannah virus–a probable new species of pestivirus–what have we found in the last 10 years? Anim Health Res Rev. (2015) 16:60–3. 10.1017/S1466252315000031 26050573

[20] Blomström AL Fossum C Wallgren P Berg M. Viral metagenomic analysis displays the co-infection situation in healthy and PMWS Affected pigs. PLoS ONE (2016) 11:e0166863. 10.1371/journal.pone.0166863 27907010 PMC5131951

[21] Muñoz-González S Canturri A Pérez-Simó M Bohórquez JA Rosell R Cabezón O. First report of the novel atypical porcine pestivirus in Spain and a retrospective study. Transbound Emerg Dis. (2017) 64:1645–9. 10.1111/tbed.12699 28941140

[22] Yuan J Han Z Li J Huang Y Yang J Ding H. Atypical porcine pestivirus as a novel type of *pestivirus* in pigs in China. Front Microbiol. 8:862. 10.3389/fmicb.2017.00862 28553280 PMC5425480

[23] Kim S Jeong C Yoon S Lee K Yang M Kim B Detection of atypical porcine pestivirus (APPV) from a case of congenital tremor in Korea. Korean J Vet Serv. (2017) 40:209–13. 10.7853/kjvs.2017.40.3.209

[24] Mósena ACS Weber MN da Cruz RAS Cibulski SP da Silva MS Puhl DE Presence of atypical porcine pestivirus (APPV) in Brazilian pigs. Transbound Emerg Dis. (2018) 1:22–6. 10.1111/tbed.12753 29119697

[25] Possatti F Headley SA Leme RA Dall Agnol AM Zotti E de Oliveira TES. Viruses associated with congenital tremor and high lethality in piglets. Transbound Emerg Dis. (2018) 65:331–7. 10.1111/tbed.12807 29322653

[26] Dessureault FG Choinière M Provost C Gagnon CA. First report of atypical porcine pestivirus in piglets with congenital tremor in Canada. Can Vet J. (2018) 59:429–32. 29606732 PMC5855290

[27] Dénes L Biksi I Albert M Szeredi L Knapp DG Szilasi A. Detection and phylogenetic characterization of atypical porcine pestivirus strains in Hungary. Transbound Emerg Dis. (2018) 65:2039–42. 10.1111/tbed.12981 30105779

[28] Williamson S Group PE Congenital tremor associated with atypical porcine pestivirus. Vet Rec. (2017) 180:42–3. 10.1136/vr.j121 28082699

[29] Postel A Meyer D Cagatay GN Feliziani F De Mia GM Fischer N. High abundance and genetic variability of atypical porcine pestivirus in pigs from Europe and Asia. Emerg Infect Dis. (2017) 23:2104–7. 10.3201/eid2312.170951 29148382 PMC5708225

[30] Zhang K Wu K Liu J Ge S Xiao Y Shang Y. Identification of atypical porcine pestivirus infection in swine herds in China. Transbound Emerg Dis. (2017) 64:1020–3. 10.1111/tbed.12659 28497656

[31] Zhang H Wen W Hao G Hu Y Chen H Qian P. Phylogenetic and genomic characterization of a novel atypical porcine pestivirus in China. Transbound Emerg Dis. (2018) 65:e202–e204. 10.1111/tbed.12675 28710801

[32] Gatto IRH Arruda PH Visek CA Victoria JG Patterson AR Krull AC. Detection of atypical porcine pestivirus in semen from commercial boar studs in the United States. Transbound Emerg Dis. (2018) 65:339–43. 10.1111/tbed.12759 29144025

[33] Shen H Liu X Zhang P Wang L Liu Y Zhang L. Identification and characterization of atypical porcine pestivirus genomes in newborn piglets with congenital tremor in China. J Vet Sci. (2018) 19:468–71. 10.4142/jvs.2018.19.3.468 29284212 PMC5974529

[34] Cagatay GN Antos A Meyer D Maistrelli C Keuling O Becher P. Frequent infection of wild boar with atypical porcine pestivirus (APPV). Transbound Emerg Dis. (2018) 65:1087–93. 10.1111/tbed.12854 29527814

[35] Colom-Cadena A Ganges L Muñoz-González S Castillo-Contreras R Bohórquez JÁ Rosell R. Atypical porcine pestivirus in wild boar (*Sus scrofa*). Spain Vet Rec. (2018) 183:569. 10.1136/vr.104824 30201807

[36] Possatti F Oliveira TES Leme RA Zotti E Dall Agnol AM Alfieri AF. Pathologic and molecular findings associated with atypical porcine pestivirus infection in newborn piglets. Vet Microbiol. (2018) 227:41–4. 10.1016/j.vetmic.2018.10.026 30473350

[37] Ronquist F Huelsenbeck JP. MrBayes 3: Bayesian phylogenetic inference under mixed models. Bioinformatics (2003) 19:1572–4. 10.1093/bioinformatics/btg180 12912839

[38] Miller MA Pfeiffer W Schwartz T Creating the CIPRES Science Gateway for inference of large phylogenetic trees. In Gateway Computing Environments Workshop (GCE). New Orleans, LA (2010). p. 1–8.

[39] Zhang H Wen W Hao G Chen H Qian P Li X. A subunit vaccine based on E2 protein of atypical porcine pestivirus induces Th2-type immune response in mice. Viruses (2018) 10:673. 10.3390/v10120673 30486487 PMC6315727

